# Aggressive Giant Cell Tumor of the Scapula in a 35-Year-Old Woman

**DOI:** 10.7759/cureus.25553

**Published:** 2022-05-31

**Authors:** Delange Hendrick Augustin, Delange Augustin, Clifford Georges Patrick Khawly, Almenord Pharol, Lerby Exantus

**Affiliations:** 1 Orthopaedics and Traumatology, Hopital Universitaire de la Paix, Port-au-Prince, HTI; 2 Radiology, Hopital de l'Universite d'Etat d'Haïti, Port-au-Prince, HTI

**Keywords:** limited resources, scapulectomy, woman, scapula, agressive giant cell tumor

## Abstract

Giant cell tumors (GCTs) are a category of benign but locally aggressive tumors that are challenging to treat. GCT topography most often affects the epiphyseal and metaphyseal regions of long bones but rarely flat bones. We report the case of a 35-year-old woman with an aggressive GCT of the right scapula. Unfortunately, the late presentation of this case to the emergency department makes the scapulectomy more difficult to carry out effectively. As this case highlights, the lack of access to primary care and the lack of available family physicians in the community remains detrimental to good medical care in systems with limited resources.

## Introduction

Giant cell tumors (GCTs) are a category of benign but locally aggressive tumors. Although benign, GCTs show a tendency for significant bone destruction, local recurrence, and occasionally metastasis [1,2]. Their topography often affects the epiphyseal and metaphyseal regions of the long bones, rarely affecting flat bones [3]. GCTs represent approximately 5% of all bone tumors with a slight female predominance and a female to male ratio of 1.40:1.28 [4,5].

Pain is the most common symptom of GCTs. Swelling and deformity are associated with larger lesions, and spreading to the surrounding soft tissue is common. The incidence of pathological fractures on presentation is 11% to 37% [6]. Bone GCTs are the most difficult benign bone tumors to treat [7]. We present a rare and challenging case of a GCT involving the right scapula in a 35-year-old woman.

## Case presentation

A 35-year-old woman was presented with apparent dyspnea. She reported pain in the right shoulder lasting five years, followed by the appearance of a mass on the inferior angle of the scapula, rapidly progressing, leading to dyspnea on exertion and causing the patient to seek consultation. On admission, her blood pressure was 140/90 mmHg, her oxygen saturation was 98%, she was afebrile, her heart rate was 77 beats per minute, and her respiration rate was 26 breaths per minute. On questioning, she reported dyspnea on exertion associated with right shoulder pain.

**Figure 1:**
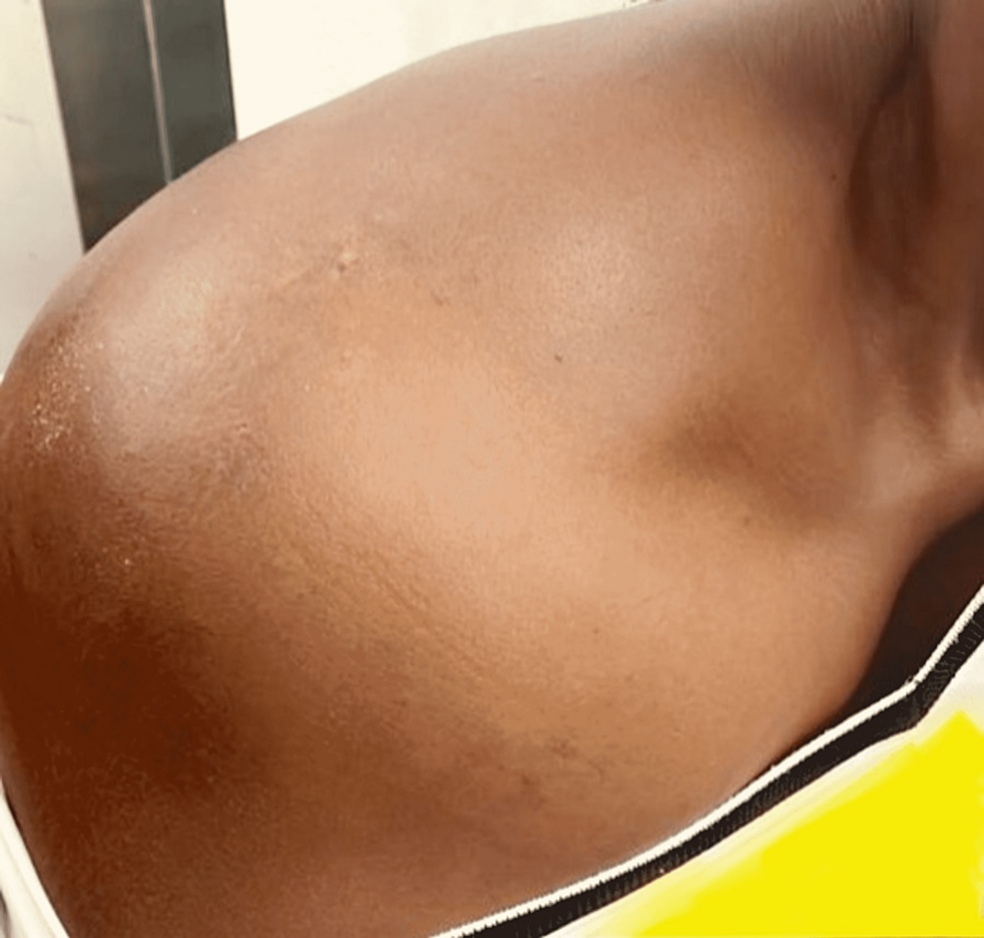
Right shoulder

The physical examination revealed diffuse swelling of the scapular region and the right shoulder (Figure 1). On palpation, the mass was hard and painless, with functional limitations of the shoulder including 80° flexion, 10° extension, 40° internal rotation, 20° external rotation, 45° abduction, and 10° adduction. The sensation of the dermatomes of the right upper limb remained intact, and we noted no evidence of motor deficit of the nerves in the region.

**Figure 2:**
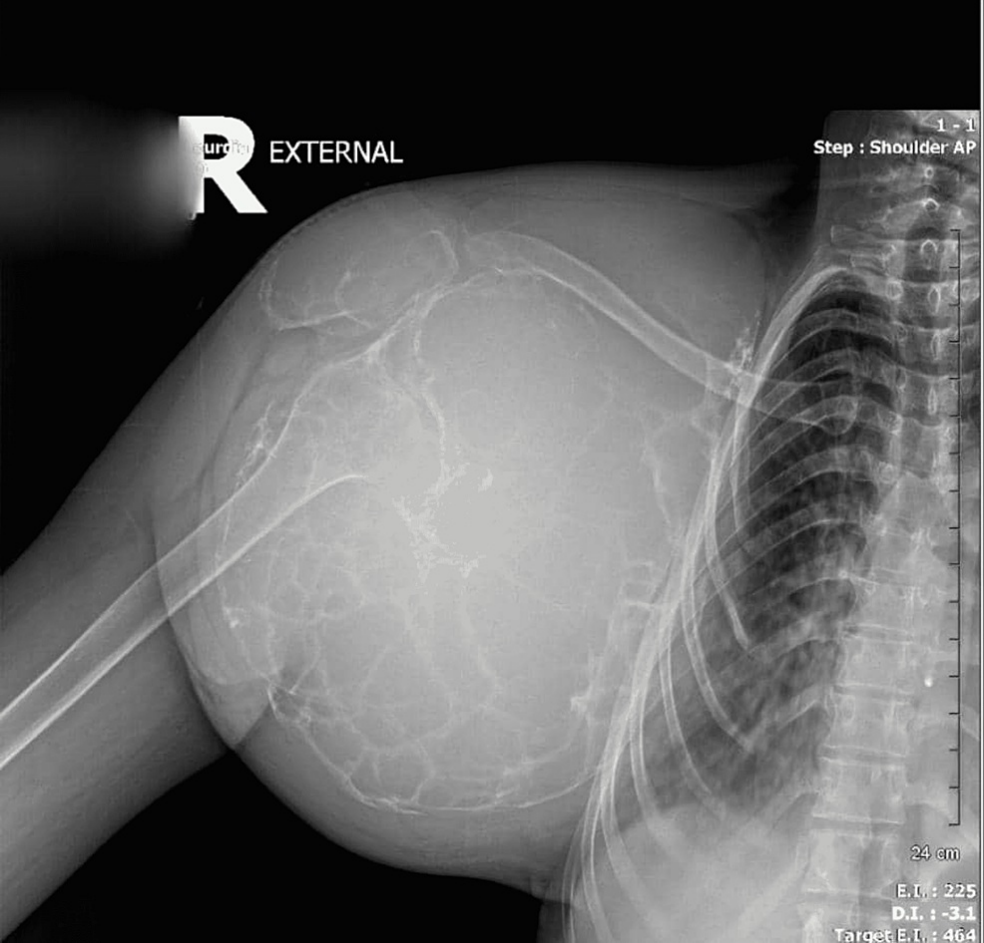
Right shoulder X-ray

**Figure 3:**
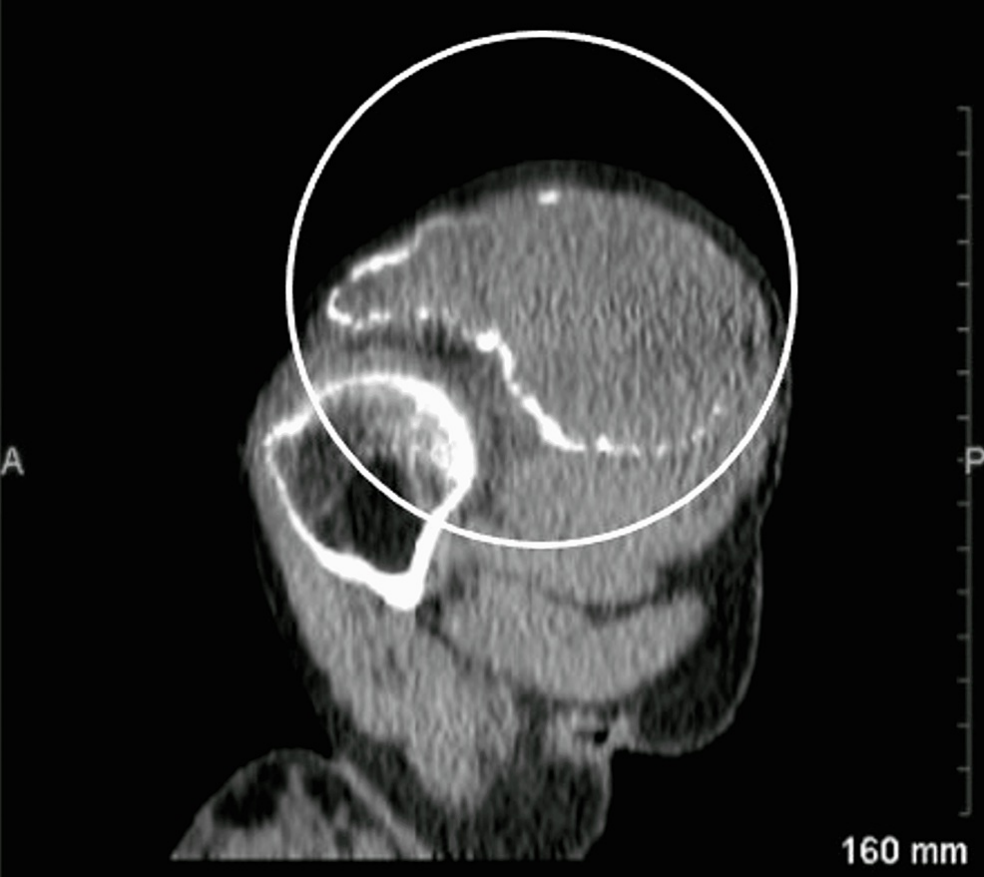
Right shoulder CT-scan

**Figure 4:**
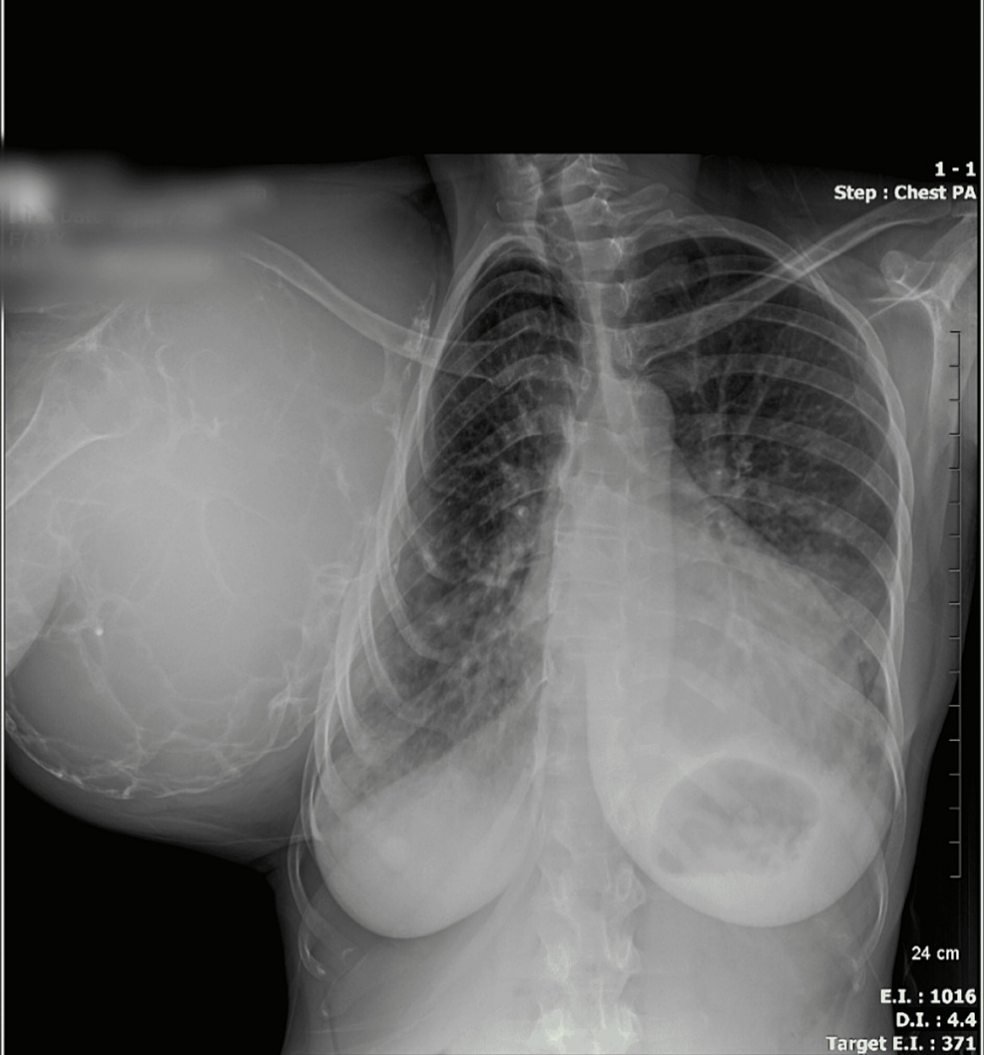
Chest X-ray

A shoulder X-ray revealed an increase in the volume of the scapula, cortical thinning, and a range of multiple lacunar osteolytic lesions with a classic soap bubble appearance, eccentric, expansile, well-defined, and involving the entire bone without intralesional matrix (Figure 2). Computed tomography revealed peripheral calcifications (Figure 3). A chest X-ray revealed compression of the right hemithorax without evidence of pulmonary metastases or alteration of the cortex of the surrounding bony structures (Figure 4). Based on the radiological findings, she was diagnosed with aggressive GCT of the right scapula.

The bone biopsy results were consistent with the radiological findings and showed many multinucleated giant cells averaging more than 70 nuclei. Round and oval uniform mononuclear cells were intermingled. The nuclei of the giant cells resemble those of mononuclear cells. We revised her diagnosis to Enneking Stage IIa aggressive GCT of the right scapula.

A total right scapulectomy would be the ideal treatment. However, after an informed discussion, the patient did not wish to proceed with this line of treatment. Instead, we opted for palliative treatment (based on chemotherapy and radiotherapy) to limit the expansion of the tumor with strict monitoring of the patient through frequent follow-up appointments.

## Discussion

Bone GCTs tend to occur in the distal femur and proximal tibia. Reports of GCT involvement in the scapula are rare in the literature [8]. The discovery of the tumor in our patient was fortuitous because her main reason for consultation was dyspnea. Our main diagnostic indicator was the classic soap bubble appearance in the standard shoulder radiograph series. As the tumor compressed the right hemithorax, the scapulectomy would not only facilitate tumor resection but also improve the dyspnea.

Various surgical options for scapular tumors have been described. Total scapular prostheses after scapula tumor resection are available, but long-term outcomes are unknown [9]. Similarly, various allografts have been described in the literature [9,10], but they are associated with graft collapse and fracture complications. The scapula is a sophisticated sesamoid bone within the shoulder girdle muscles [11]. Thus, after scapulectomy, if the epimysium is reconstructed and the glenoid fossa remains undisturbed, proper shoulder function can be expected. The functional outcomes depend on the preservation of the glenoid fossa and the reconstruction of the muscles around the shoulder, namely the deltoid and trapezius. Even after total scapulectomy, upper limb function can be preserved because it is centered mainly around the elbow and the hand [12]. While we may not be able to preserve the glenoid cavity, the treatment of choice for the patient would be a total scapulectomy with a muscular reconstruction of the trapezius and deltoid with the intent that the elbow and hand function postoperatively. Radiation therapy and embolization are generally reserved for cases where surgical treatment is not feasible [13]. Faced with the patient's nonadherence to surgical care, we opted for palliative care based on chemotherapy and radiotherapy.

In June 2013, the United States Food and Drug Administration approved denosumab as an alternative treatment for unresectable cases in adults and skeletally mature adolescents. In fact, denosumab provides favourable and consistent clinical and radiographic responses, which facilitates less aggressive surgical treatment, especially joint preservation. However, the local recurrence rate for GCTs of bone following resection does not seem to be affected by denosumab and remains a concern [14,15].

Indeed, despite the aggressive character of the tumor and its grading and staging, less than 25% of these cases carry a risk of metastasis [16], and the overall prognosis is generally good. However, lung metastases have been cited as the cause of death in 16% to 25% of reported cases [17,18]. In their study on the risks of pulmonary metastasis from GCTs of bone, Chan et al. reported an increased risk of pulmonary metastasis in younger patients who present with Enneking stage III disease, develop a local recurrence, and/ or present with axial disease. The mode of treatment was not associated with the development of lung metastases [19].

## Conclusions

GCTs are relatively common bone tumors and are generally benign. They usually originate from the metaphysis of long bones, extend to the adjacent epiphysis on the articular surface, and have a narrow transition area. Although rare, this case describes an aggressive form of the tumor located on the scapula of a 35-year-old woman. The late presentation of this case to the emergency department makes the scapulectomy more difficult to carry out effectively. As this rare case highlights, the lack of access to primary care and a lack of available family physicians in the community remains a detriment to good medical care in systems with limited resources.
